# Large-Scale Survey of Unselected Automated Visual Fields in a Major Reading Center: Patterns and Data Analysis

**DOI:** 10.6064/2012/127562

**Published:** 2012-05-20

**Authors:** Lilly Zborowski-Naveh, Rita Ehrlich, Moshe Luski, Dov Weinberger, Mona Boaz, Dan D. Gaton

**Affiliations:** ^1^Deptartment of Ophthalmology, MOR Institute for Medical Data, 51108 Bnei Brak, Israel; ^2^Department of Ophthalmology, Wolfson Medical Center, 58100 Holon, Israel; ^3^Department of Ophthalmology, Rabin Medical Center, Beilinson Hospital, 49100 Petach Tikva, Israel; ^4^Sackler Faculty of Medicine, Tel Aviv University, 69978 Tel Aviv, Israel; ^5^Department of Epidemiology, Wolfson Medical Center, 58100 Holon, Israel

## Abstract

A prospective, randomized study was conducted to survey a large number of automated perimetry examinations in a central reading institute, obtaining practical information on unselected referred patients and their clinician “consumers”. Visual field records of 1041 patients were obtained, each evaluated by one of three glaucoma specialists. Statistical analysis was applied on demographics, physician characteristics, test reliability and visual field scores. Reliability was scored on a scale of 1 (excellent) to 5 (uninterpretable). Data from earlier examinations of these patients was also analyzed. The large majority of patients (70.4%) were referred due to glaucoma, ocular hypertension or suspected glaucoma. Most of the patients had threshold strategies: FastPac 24-2 or 30-2 (88.9%), Full Threshold (0.7%), and 10-2 (0.5%). In only 7 patients was short-wavelength automated perimetry (SWAP) performed. The Swedish Interactive Testing Algorithm (SITA) was applied in 1.0% of cases. More than half (56.8%) of the population had a reliability score of 1, and 22.7% had a score of 2, indicating a valid result for 79.4% of patients, providing clinically useful information. Linear regression analyses indicated that the Mean Defect was a better predictor of the visual field score than the Corrected Pattern Standard Deviation (CPSD), for the entire group and for each visual field score subgroup.

## 1. Introduction

 Visual field evaluation is critical in glaucoma and neuro-ophthalmic diagnosis and followup. All the major studies of the last decade or so including EMGT [1], AGIS [2], OHTS [3], and CIGTS [4] have used visual field criteria to establish eligibility and for followup. Each of those studies created their own scoring system and their criteria for defining progression. In all cases the reliability requirements were high, and the patients needed to be available for fairly frequent exams.

 In the real world of clinical practice our patients are often less than perfect candidates, they may not return exactly on schedule for their exams, and the community-based ophthalmologists may not all be “up to speed” on perimetry interpretation. The study scoring systems are not always available or practical for everyday clinical use.

 In our country, most visual field examinations are referred to diagnostic centers. The Mor Institute provides comprehensive state-of-the-art diagnostic services to 500,000 patients per year. It receives referrals from about 150 ophthalmologists nationwide and performs more than 10,000 perimetry examinations annually. One of three glaucoma specialists reads the printouts and returns a written evaluation to the referring physician.

 We sought to utilize this large group of unselected patients' examinations to gain insight into real everyday practice and to determine how to improve test results and patient care as well as physician practice patterns.

 To the best of our knowledge, no similar survey of this scale has been reported to date.

## 2. Materials and Methods

 The present prospective study included all automated perimetry examinations conducted at the Mor Institute during one month (August 2002). The participating technicians were experienced and have been trained to both monitor fixation and encourage the patients during testing. The printouts were read by three of the authors (L. Zborowski-Naveh, M. Lusky, and D. D. Gaton). The readers evaluated the printout as usual and sent a letter with the results to the referring physician. They then completed the survey questionnaire. The right eye or the only eye of each patient was included in the survey.

 The survey queried four categories of information: patient-related characteristics (age, gender, and referring diagnosis); physician-related characteristics (test algorithm and test target size requested); test results (reliability score, reasons for loss of reliability, visual field score, mean deviation, and corrected pattern standard deviation); and longitudinal data, where applicable (number of previous examinations, change in reliability score, and change in visual field score).

 Reliability was scored on a scale of 1 (excellent) to 5 (uninterpretable). The reader assigned a score on the basis of four parameters: fixation loss; false-positive errors; false-negative errors; white scotomas. For any field with a reliability score greater than 1, the reason was cited as any of the above, alone or in combination.

 A linear visual field scoring system was designed to correspond to practical clinical usage. The criteria for score assignment are provided in Table 1.

### 2.1. Data Analysis

 Data were analyzed with the SPSS for Windows, version 15.0.1 (SPSS Inc., Chicago, IL, USA). For continuous variables (age and visual field score), descriptive statistics were calculated and reported as mean ± standard deviation. Categorical variables (specific diagnosis and patient gender) were described using frequency distributions. Continuous variables were compared by reader and by age group using one-way analysis of variance (ANOVA). Continuous variables were compared by diagnostic group (glaucoma versus ocular hypertension) and gender using *t*-test for independent samples. Associations between continuous variables and age were analyzed with Pearson's correlation coefficients. Previous test results were compared to present results using *t*-test for paired samples. All tests were two-sided and considered significant at *P* < 0.05.

## 3. Results

 During the study period, 1041 visual field records were obtained.

### 3.1. Patient-Related Data

 Mean age of the surveyed population was 61.87 (±17.10) years (range 6–93). Only 18.2% were younger than 50 years; 70.8% were aged 50 to 79 years, and 11.9% were aged 80 years or more.

 Significant differences were noted in the proportion and age of men and women referred for testing. Women comprised 56.6% of the survey population and were of mean age 59.95 (±17.42) years. Men comprised 43.4% of the population and were of mean age 64.32 (±16.38) years. There were more women than men in each decade of life except the ninth (*P* = .047).

 The distribution of referring diagnoses by number of patients and mean age is shown in Table 2. The large majority of patients (70.4%) were referred for reasons related to glaucoma; known glaucoma, ocular hypertension (OHT), or suspected glaucoma. There was a significant difference in mean patient age among the diagnostic groups (*P* < 0.001), with the youngest patients referred mainly for neurology/headache and myopia. Clinically, the most significant variation was the 5-year difference between the glaucoma and the OHT/suspected glaucoma groups (*P* < 0.001). Forty-two of the 395 patients (10.6%) with OHT/suspected glaucoma were aged 50 years or less and an additional 5 were aged 51 to 55 years.

### 3.2. Physician-Related Data

 The referring physician specified the test algorithm to be used. Most of the patients were tested with threshold strategies: FastPac 24-2 or 30-2 (88.9%), full threshold (0.7%), and 10-2 programs (0.5%). The 3-zone 120-point screening program was specified by 6.7% of physicians, and the macula program with red and white targets, by 1.1%. In only 7 patients short-wavelength (blue-yellow) automated perimetry (SWAP) was performed; 6 of them had OHT/suspected glaucoma and were aged 55 years or less. The Swedish interactive testing algorithm (SITA) was applied in 1.0% of cases.

### 3.3. Test Results

#### 3.3.1. Reliability

 The mean reliability score was 1.68. There was a small but statistically significant difference in mean score between men (1.71) and women (1.66) (*P* < 0.001).

 The tests of more than half (56.8%) the survey population had a reliability score of 1, and 22.7% had a score of 2, indicating a valid result for 79.4% of patients. An additional 15.8% had a reliability score of 3 which can at times still provide clinically useful information.

**Figure 1:**
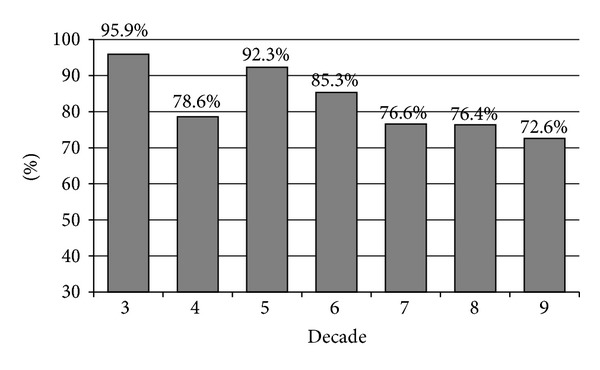
Percent of patients in each decade of life with a reliability score of 1 or 2.

 Reliability was correlated with patient age. When children were excluded, we found a steady decrease in the proportion of “excellent” reliability test scores (score 1 by age group), from 91.7% for patients in the third decade to 47.1% for patients in the ninth decade (*P* < 0.001). The decrease in reliability with age was sustained when we grouped patients with test scores 1 or 2 together. Nevertheless, even in the over-80-year group, 72.6% of patients had a valid test result (Figure 1).

 The most common reason cited by the readers for low test reliability was loss of fixation (90.3% of cases), followed by false-negatives (25%) and false-positives (7.0%).

#### 3.3.2. Visual Field Score

**Figure 2:**
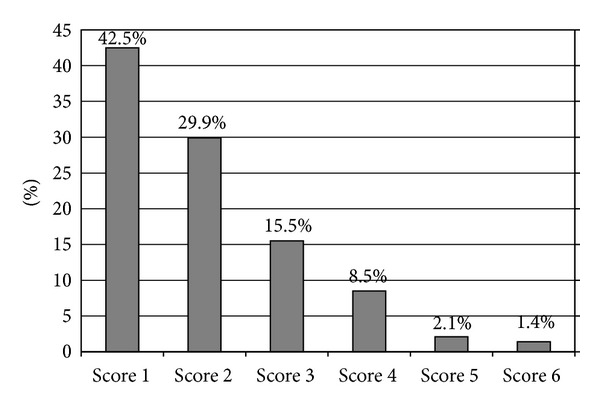
Distribution of visual field scores.

 Each field was assigned a damage score, and each of the glaucoma specialists (L. Zborowski-Naveh, M. Lusky, and D. D. Gaton) read about one-third of the fields. No statistically significant difference was found among the mean scores of the 3 readers (Table 3). The mean score for the entire group was 2.01. The distribution of visual field scores is shown in Figure 2.

Table 4 summarizes the mean visual field scores by diagnosis. No specific complaints or diagnoses were noted in 109 (10.5%) patients. The myopia group (which was also the youngest) had the highest score, and the hydroxychloroquine group the lowest. Although the difference in mean score between the glaucoma group (2.28) and OHT/suspected glaucoma group (1.92) was not large, it was statistically significant (*P* < 0.001).

#### 3.3.3. Correlation of Visual Field and Reliability Scores

 The visual field score was positively correlated to the reliability score (*r* = 0.36): the lower the reliability, the greater the chance of a defective visual field.

#### 3.3.4. Mean Deviation (MD)

 The mean MD for the 935 size III threshold tests was −3.30 dB (±3.57 dB). The mean MD in the group of patients aged 40 years or less was −3.68 ± 4.4 dB. However, from the fifth through the ninth decade, there was a steady decrease in MD, from −2.3 dB to −4.24 dB (*P* < 0.001).

#### 3.3.5. Corrected Pattern Standard Deviation (CPSD)

 The mean CPSD for the 935 size III threshold tests was 1.94 dB. A nonsignificant trend toward an increase in CPSD with age was observed.

 There was a significant correlation of the MD with the CPSD (*r* = .27, *P* < 0.001), and of both the MD and the CPSD with the visual field score (*r* = .55, *P* < 0.001 and.54, *P* < 0.001, resp.). Linear regression analyses indicated, however, that the MD was a better predictor of the visual field score than the CPSD, for the entire group and for each visual field score subgroup.

### 3.4. Longitudinal Data

 A previous visual field test was available for comparison for 608 of the 1041 patients (58.1%) (Table 5). Analysis of the number of patients in whom the disease progressed yielded a significant difference between the glaucoma and the OHT/suspected glaucoma groups (25% versus 15.8%, resp., *P* < 0.012).

In 505 of the 608 cases, the previous or the first in a series of visual field exams was available. The current mean reliability score for this subgroup was 1.75. The mean reliability score assigned to the earlier visual field test was 1.63. This difference was not statistically significant, suggesting the absence of a “learning effect” for reliability.

## 4. Discussion

 This survey is unique in the literature of perimetry in that it included a very large population of entirely unselected cases. Our purpose was to extract useful clinical information that could be applied to daily practice. All other studies were either limited to small groups without pathology [5] or selected study groups with exclusion of unreliable fields [1–7].

 The finding that glaucoma was the main reason for the test (70%) was expected. The progressive increase of the MD with age has also been described in the literature [5]. Other results were less obvious.

### 4.1. Demographics

 Female patients accounted for 56.6% of the whole population; however, this rate is only slightly higher than the proportion of women in the general population (53.9% for those 45 years old or more) [8]. The mean age of the women was significantly lower than that of the men (59.95 and 64.32 years, resp.), which may reflect an actual gender-related difference in the age distribution of patients with these pathologies. Some of the earlier population-based studies of the incidence or prevalence of glaucoma [9–11] did not show any gender- or age-related difference, whereas others reported a higher prevalence in men [12–14]. Only in the Hispanic population in the United States [15] was a higher prevalence noted in women.

 Our finding may also be explained by a bias in diagnostic referrals or a difference in utilization of primary health care services. The possibility that the difference is a result of chance cannot be entirely discounted either. This question merits further study.

 The older mean age (by more than 5 years) of the glaucoma group than the OHT/suspected glaucoma group, combined with the significantly higher visual field damage score in the patients with glaucoma, supports the notion that glaucoma is preceded either by OHT or by suspicious disc changes.

### 4.2. Algorithm Requested

 The low rate of requests for the SITA program (1%) suggests a certain degree of conservatism among our referring physicians and highlights an important area in which educational activities may be applied.

 The SWAP program was used in only 7 patients, although we identified 47 patients with OHT or suspected glaucoma who were 55 years old or less at the time of examination. SWAP testing can detect visual field damage earlier than standard white on white perimetry, [16] and it is particularly suitable for younger patients because of their low incidence of nuclear sclerosis. Yet it was not applied in most of the patients in our population who might have benefited from it, maybe due to lack of clinician awareness at the time of patient recruitment to the study.

### 4.3. Reliability and Visual Field

 About 80% of our random, unselected population were able to cooperate well enough for the clinician to obtain a reliable field (Figure 1). Test reliability in an additional 15% was rated intermediate, that is, sufficient for the clinician to obtain partially useful, if not entirely accurate, information. Even the group of patients in the ninth decade of life had a 72.6% rate of valid field results. These data suggest that clinicians should not hesitate to test the elderly with automated perimetry.

 The ocular hypertension treatment study (OHTS) [3] reported better reliability results than ours with 79% of patients having reliable fields by strict criteria and 97% by slightly more liberal criteria, corresponding roughly to our level 1 and 2. Although the trend in our study was similar, the difference in reliability was probably attributable to the more restricted patient population in the OHTS.

 Like in the OHTS, the most common reason for loss of reliability in our study was fixation loss. Accordingly, reliability correlated with the visual field score. These findings suggest a confounding effect of reliability on test results. Therefore, focusing efforts on improving fixation of the examinees will result in better reliability and “cleaner” test results.

 Patients with advanced field loss may find it difficult to maintain fixation, and even slight movements at the margins of deep scotomas can easily produce false-negative results. This might explain some of the shared variability in reliability and damage. In addition, a true decrease in reliability can be the cause of a falsely poor result, producing a confounding effect. Therefore, it is important to improve reliability for each examination, especially in patients with advanced field loss.

 Previous studies have described a learning effect of repeated visual field testing on test results, even when the initial reliability was good [17–19]. Our data, however, do not support a specific learning effect. In general, reliability or lack thereof persisted from the first test to the later one. We do not suggest that repeated attempts should not be made to improve test reliability, only that these attempts may not always succeed as intended. Learning effects may be stronger if the examinations are closely spaced in time. Further analysis of the time elapsing between repeated examinations should be made in future studies.

 The MD and the CPSD both correlated with the visual field score, but the MD was a better predictor. This is contrary to our expectations of a lower predictive value of the MD, given its greater sensitivity to lens changes or refractive errors than the CPSD [10, 20], at least at the level of early to moderate field damage.

### 4.4. Implications for Physicians

 This study was not designed to evaluate the criteria of the community physicians for requesting perimetry. However, the fact that 42.5% of the fields were normal might suggest that the “threshold” for referral was fairly low and that a healthy degree of suspicion was being maintained.

 In our survey, the referring physician assigned the diagnosis; consequently, there was no standardization of the diagnostic criteria or diagnostic confirmation. Nevertheless, we found a clear and significant difference in the mean visual field score between the glaucoma group (2.28) and the OHT/suspected glaucoma group (1.92), in addition to a significantly higher rate of deterioration in the glaucoma group (25% versus 15.8%). The findings were not necessarily confirmed by repeated fields, but the statistics of progression could imply that more aggressive treatment of glaucoma in the community is needed. This is consistent with the results of the Advanced Glaucoma Intervention Study (AGIS) [21] and others [13, 22], which demonstrated a benefit for consistently lower pressure in preventing further field loss in patients with glaucoma.

## 5. Summary

 In summary, we describe the results of a large-scale survey of unselected patients undergoing automated perimetry. We were able to gain significant insight into reliability according to patient age and, perhaps, the behavior of disease progression in this group of patients as well as into visual field scores according to referring clinical diagnosis. In addition, the actual practice patterns of a large and representative group of ophthalmologists were delineated.

 We were able to gain insight into the patterns of physician referral and learn where to focus additional education for better utilization of perimetry options. Most patients are capable of giving a test result reliable enough to be clinically useful even in the elderly age bracket. The need for more aggressive treatment of glaucoma patients is implied in the proportion of cases with progression. 

## Figures and Tables

**Table 1:** Visual field scoring system.

Score	Degree of damage	Description
1	No damage	Normal visual field
2	Mild damage	Enlarged blind spot, slight nasal or temporal depression, mild peripheral constriction, and mild diffuse or nonspecific changes
3	Moderate damage	Changes in one quadrant or moderate nasal depression, relative central, or cecocentral scotomas
4	Advanced damage	Changes in two quadrants above or below the midline or advanced nasal depression. Deep central or cecocentral depression
5	Severe damage	Significant defects above and below the midline
6	End stage	Tubular field with or without a temporal island

**Table 2:** Distribution of referring diagnoses by mean age.

Diagnosis	*N* (%)	Mean age (years)
Glaucoma	337 (32.4)	68.8
OHT/suspected glaucoma	395 (38)	63.2
Retinal pathology (except myopia)	29 (4.0)	61.5
Neurologic/headache	160 (15.4)	47.9
Myopia	8 (0.8)	46.4
Cataract	2 (0.2)	83.1

*P < 0.001.

OHT: Ocular hypertension.

**Table 3:** Mean visual field scores of the 3 readers.

Reader	*N*	Mean score^∗^
1	348	1.9827 ± 1.2875
2	347	2.0870 ± 1.1042
3	346	1.9681 ± 1.0711

*P ≤ 0.340.

**Table 4:** Mean visual field score by diagnosis.

Diagnosis	*N*	Mean visual field score
Myopia	8	2.38
Retinal-vascular	17	2.29
Glaucoma	333	2.28^∗^
Neurologic/headache	158	2.17
Macular changes	12	1.92
OHT/suspected glaucoma	392	1.92^∗^
Hydroxychloroquine	12	1.58

*P < 0.001.

OHT: ocular hypertension.

**Table 5:** Rates of progression/stability when previous fields were available.

	Entire group	Glaucoma	OHT/Suspected glaucuoma
	*N* (%)	*N* (%)	*N* (%)
Stable	463 (76.5)	176 (71)	184 (82.8)
Progressed	116 (19.2)	62 (25)^∗^	35 (15.8)^∗^
Improved	22 (3.6)	10 (4)	3 (1.4)
Indeterminate	7 (0.7)		

*P < 0.012.

OHT: ocular hypertension.
